# Social Factors as Modifiers of Hurricane Irene Evacuation Behavior in Beaufort County, NC

**DOI:** 10.1371/currents.dis.620b6c2ec4408c217788bb1c091ef919

**Published:** 2013-06-05

**Authors:** Kristen Ricchetti-Masterson, Jennifer Horney

**Affiliations:** Gillings School of Global Public Health, University of North Carolina at Chapel Hill, Chapel Hill, North Carolina, USA

## Abstract

Encouraging residents in high-risk areas to evacuate before a hurricane makes landfall is one of the few ways to reduce hurricane-related morbidity and mortality. However, demographic factors associated with evacuation in at-risk groups have not been consistent across studies. To determine if social factors (social control, social cohesion, and social capital) modified the relationship between demographic groups and failure to evacuate from Hurricane Irene, the authors conducted a cross-sectional stratified two-stage cluster sample among residents of Beaufort County, NC. Of 226 attempted rapid response interviews, 205 were completed (response rate = 90.7%). Data were analyzed using generalized linear modeling, which produced crude risk differences to estimate the association between failure to evacuate from Hurricane Irene and a number of demographic and social factors; effect measure modification (EMM) was assessed on the additive scale through stratified analyses of key social factors. There were no significant associations between demographic or social factors and evacuation in the bivariate analysis. However, EMM was present for households with high social capital or social cohesion among special needs residents, those over age 65, males, and non-whites. In Beaufort County, NC, future hazard mitigation plans should include evacuation messages tailored for households with high social capital or social cohesion.

## Introduction

**Hurricane evacuation behavior**

Encouraging residents in high-risk areas to evacuate before a hurricane makes landfall is one of the few ways to reduce hurricane-related morbidity and mortality. However, many factors have been shown to discourage an individual’s decision to evacuate. In light of this problem, public health interventions must increase rates of evacuation, especially among high-risk groups.

Studies have consistently shown that those who have lived through previous hurricanes without major harm are less likely to evacuate, as are those who believe that his or her home is not in a high-risk location for hurricane damage or that the storm is not severe enough to warrant evacuation 1 ^,^ 2 ^,^ 3 ^,^ 4. As Baker succinctly states, “Most residents who feel unsafe staying where they are during a storm tend to leave, and those who feel safe tend to stay,” 1 (p. 293).

Yet safety considerations are not the only influence on an individual’s decision to evacuate. Individuals with limited access to transportation and additional funds to purchase fuel, food, lodging, etc. are less likely to evacuate prior to a hurricane 1 ^,^ 5. Opting to stay behind to protect personal property is another reason cited for refusing to evacuate, as is having to stay for perceived community, occupational, and family obligations 1 ^,^ 3. In addition, studies have shown that demographic factors, such as gender, education, race/ethnicity, age, disability status, income, home ownership, pet ownership, the presence of child residents, and the presence of elderly residents have been linked to evacuation choices 1 ^,^ 2 ^,^ 4 ^,^ 5 ^,^ 6 ^,^ 7 ^,^ 8 ^,^ 9. However, associations between evacuation in these at-risk groups have not been consistent across studies.

One proposed explanation for this inconsistency is that social factors – such as social capital, social cohesion, and social control 10 – modify the relationship between other risk factors and evacuation behaviors 11. This could have important implication on which groups are targeted for tailored interventions as part of the planning process.

**Hurricane Irene**

Hurricane Irene began over the Atlantic Ocean on August 20, 2011. It progressed across Puerto Rico and the Caribbean, where it strengthened to a Category 3 hurricane (winds up to 129 mph). It made landfall in North Carolina as a Category 1 hurricane (winds up to 95 mph) on August 27, 2011, and progressed along the eastern coast of the United States and into Canada for two days, where it was downgraded to a tropical storm. Hurricane Irene was responsible for at least 43 deaths in the US and Canada. It also caused 6.25 million people to lose power and thousands of flights to be cancelled. 12

On August 25, 2011, the President of the United States declared Hurricane Irene a federal emergency. On August 28, 2011, Beaufort County, NC, was added to the list of counties included in the federal declaration, making the county eligible for recovery funds and assistance from the Federal Emergency Management Association 13. As of March 1, 2012, North Carolina had received more than $160 million in disaster assistance for Hurricane Irene recovery efforts 14.

On August 25, 2011, the Beaufort County commissioners declared a State of Emergency, which resulted in a mandatory evacuation for all households in the 100-year flood plain and residents living in mobile homes. All other residents were under a voluntary evacuation order. 15

**Beaufort County, North Carolina**

****According to the 2010 Census, Beaufort County, NC, had 47,759 residents and 19,941 occupied households in 2010. In addition, there were also 4,747 housing units considered “vacant” by the US Census Bureau that were occupied for less than half of the year. Though some of these homes were truly unlived in, perhaps even abandoned, this category also includes vacation homes and rentals, particularly along the coast. Permanent residents of Beaufort County were 68.2% white, and 87.2% had lived in the same home for one year or longer. Just 6.3% of residents spoke a language other than English at home. Over 4 in 5 adults were high school graduates (81.5%). The median household income was $40,653, and 17.2% of the population was below the poverty level. 16

## Methods

**Sampling**

We used a stratified two-stage cluster sampling method to select 210 weighted random starting points for door-to-door interviews in Beaufort County, North Carolina 17 (Figure 1). Environmental Systems Research Institute ArcGIS 10.0 mapping software and census data were used to select the clusters and interview points (Environmental Systems Research Institute, Redlands, CA).

**Map of 30 sampling clusters in Beaufort County, North Carolina.**
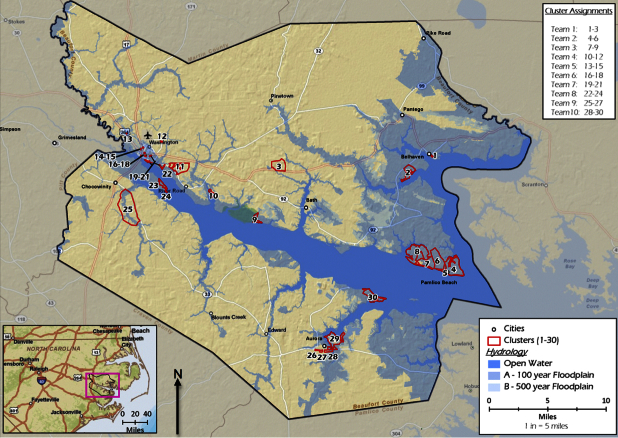

In the first stage of sampling, 30 census blocks – or clusters – were selected without replacement. Only census blocks with 10 or more households were eligible to be selected. In the first stratum, 10 clusters were randomly selected from the highest flood risk areas, among census blocks with greater than 95% of the block in the 100-year flood plain; 138 census blocks in Beaufort County met this criterion. In the second stratum, 5 clusters were randomly selected in areas with high flood risk, where greater than 50% but less than 95% of the census block is in the 100-year flood plain; 93 census blocks in Beaufort County met this criterion. In the third stratum, 5 clusters were randomly selected from the moderate to low flood risk areas, from census blocks with less than 50% of the block in the 100-year flood plain; 343 census blocks in Beaufort County met this criterion. Another 10 blocks were deliberately chosen in areas known to have experienced heavy flood damage. Within each of the strata, blocks were randomly selected with a probability proportional to the number of households.

In each cluster, we used ArcGIS 10.0 software to assign seven random GPS coordinates as interview starting points. Interview teams begin at each of the seven points in a cluster and went door-to-door until an interview was completed or all households in the cluster were exhausted, either because they had already completed an interview, were not available after multiple attempts, or refused to participate.

This sampling strategy ensures a representative analysis of the entire county with a 95% confidence interval of plus or minus 10% for each question 18.

This study was reviewed by the University of North Carolina Institutional Review Board and was deemed exempt on September 13, 2011 (study number: 11-165).

**Participation criteria**

The only requirements for participation in the questionnaire were that the respondent was over age 18 and residing in the area when Hurricane Irene made landfall.

**Data collection**

Door-to-door interviews were conducted on Friday, October 7 and Saturday, October 8, 2011, from approximately 10 AM to 6 PM each day. These dates and times were selected to maximize the opportunity to find residents at home, despite varying work and social obligations. A group of 14 volunteers from UNC’s Team Epi-Aid and 4 staff members from the UNC Center for Public Health Preparedness collected data in two-person teams using handheld GPS-enabled computers running ArcPad 10.0 geographic information systems software. The survey instrument contained 21 questions for the participant and four questions for the interviewer to answer about each respondent and his or her home (Appendix II).

**Outcome assessment**

The outcome of interest was failure to evacuate from Hurricane Irene. This variable was assessed based on answers to the question “Did you evacuate during Hurricane Irene?” Interviewers were trained to probe any unclear responses to clarify that evacuation was defined as anyone in the household choosing to temporarily re-locate from a primary residence prior to landfall of Hurricane Irene on August 27, 2011.

**Demographic assessment**

Each respondent was asked his or her year of birth to calculate age, which was dichotomized around the median of 59 years old in the analysis. Respondents were also asked to report the presence of any of the following types of residents in the home: 65 years old or older, under 18 years old, or residents with special needs that would make it more difficult to evacuate. The interview team assessed the race/ethnicity (white vs. non-white) and gender of the respondent. They also assessed the home type (single-family, apartment, or mobile home).

**Social control /cohesion/capital assessment**

We measured social control using a composite scoring system based on the responses to two statements: “I would intervene if I saw children in my neighborhood or community destroying property such as spraying graffiti or knocking down mailboxes,” and “I would intervene if I saw looting after a hurricane or other disaster.” Each question could be answered as “totally true,” which was given a score of three; “somewhat true,” which was given a score of two; “somewhat false,” which was given a score of one; or “totally false,” which was given a score of zero. The scores for each question were summed to create the composite score for social control. We dichotomized the social control score around the median to determine exposure status.

We assessed social cohesion in two different ways: using a composite score based on the participant’s responses to two statements and a score based on the number of markers of territoriality seen at the participant’s home 1 ^,^ 2 ^,^ 4 ^,^ 6 ^,^ 7 ^,^ 8 ^,^ 9 ^,^ 19.

We based the social cohesion score on responses to two statements: “People who live in this neighborhood or community are willing to help their neighbors,” and “People in this neighborhood can be trusted.” As with the social control scoring system, answers were scored on a 3-point scale ranging from totally false (0 points) to totally true (3 points). We summed the scores for these questions to create the composite social cohesion score and dichotomized around the median to determine exposure status.

For the measure of social cohesion using markers of territoriality, interviewers looked for five specific indicators of territoriality at each property: a name on the mailbox, “no trespassing” sign, fenced yard, “beware of dog” sign, and alarm system warning sign. We assigned one point for each item present at a property and totaled these points to create the markers of territoriality score. We dichotomized this score around the median to determine exposure status.

We assessed social capital in three different ways, by: belonging to organizations, the number of social interactions (including saying “hello” and visiting), and volunteering to assist with Hurricane Irene recovery activities.

To assess the organization component of social capital, respondents were asked: “Are you a member of any of the following organizations: community service (such as volunteer fire or rescue), civic group (such as Lions, Kiwanis, Rotary, etc.), veterans (such as VFW, Knights of Columbus, etc.), church, or other groups?” We assigned one point for each organization and totaled these points to create the social capital organization score, which we dichotomized around the median to determine exposure status.

We measured the presence of social capital as a result of social interaction through two different questions: “How many people do you say ‘hello’ to in a day?” and “How many friends and family do you enjoy visiting with on a regular basis?” We considered anyone who experienced greater than the median for either of these questions exposed to social capital due to social interactions.

For a measure of social capital based on volunteerism, respondents were asked, “Have you done any volunteer work to help those impacted by Irene?” We considered those who responded “yes” exposed to social capital due to volunteerism.

**Evacuation Order Knowledge**

Participants were asked, “Did an evacuation order cover your home?” Those who recalled an evacuation order for his or her home were asked, “Was this evacuation order voluntary or mandatory?”

**Data analysis**

Data analysis was conducted using SAS 9.2 software (SAS Institute, Cary, NC). We used generalized linear models to produce crude risk differences (RDs) for the bivariate association between hurricane evacuation and all demographic and social variables. The 95% confidence intervals (CIs) for RDs were calculated using a continuity correction for the Wald asymptotic confidence limits, which correctly approximates the confidence limits for a binomial outcome 20.

We assessed effect measure modification (EMM) on the additive scale through stratified analyses. Strata-specific RDs and 95% CIs were calculated for all main demographic and social variables. Stratum-specific CIs that did not include the other stratum’s point estimate were explored further, as were stratified results with estimates on opposite sides of the null value.

## Results

Interview teams approached 472 homes, and 226 had an eligible resident at home (contact rate of 47.9%). Of the 226 attempted interviews, 205 were completed (response rate of 90.7%). The completion rate for this study is 97.6% (205 completed interviews out of 210 possible interviews for the sampling method). All of the 205 households that completed the interview are included in the analysis.

**Study population demographics**

Among the 205 respondents, most were white (72.2%), female (63.9%), over 45 years old (80.0%), lived in a single-family home (65.9%), and owned their home (75.6%) (Table 1). The average year of birth for respondents was 1953 (58 years old), with a median of 1952 (59 years old). Respondents reported living in their current residence for an average of 15 years and a median of 10 years. Nearly two in five homes reported a resident who is 65 years old or older (39.5%), 21.0% reported a resident under 18 years old, and 15.6% had a resident with special needs (Table 1). Aside from female gender, median age, and the presence of residents 65+ years old, the proportions of all key demographic variables measured in this study were within plus or minus 10% of the 2010 US Census findings for Beaufort County, NC 16.

None of the demographic factors produced statistically significant RD estimates for failure to evacuate, though having a resident with special needs was nearly significant at an alpha of 0.05 (RD = 0.19; 95% CI: -0.01, 0.39) as was having a resident under the age of 18 (RD = 0.15; 95% CI: 0.00, 0.29).

**Table 1. Study population demographics and the risk of failing to evacuate from Hurricane Irene in Beaufort County, North Carolina (n=205).** 

	**Study Population**			**Evacuated**			**Did Not Evacuate**			**Risk Difference (95% Confidence Interval)**
	n	%		n	%		n	%		
**Age**										
Born after 1952	93	45.37		24	25.81		69	74.19		0.04 (-0.09, 0.17)
Born before 1953	111	54.15		33	29.73		78	70.27		REF
Missing	1	0.49								
**Race/Ethnicity**										
White	148	72.2		44	29.73		104	70.27		REF
Non-white	56	27.32		12	21.43		44	78.57		0.08 (-0.06, 0.23)
Missing	1	0.49								
**Gender**										
Female	131	63.9		34	25.95		97	74.05		REF
Male	73	35.61		22	30.14		51	69.86		-0.04 (-0.18, 0.10)
Missing	1	0.49								
**Home Ownership**										
Rent home	48	23.41		12	25.00		36	75.00		REF
Own home	155	75.61		45	29.03		110	70.97		-0.04 (-0.18, 0.12)
Other	2	0.98								
**Home Type**										
Multi-unit or mobile home	69	33.66		15	21.74		54	78.26		REF
Single-family	135	65.85		41	30.37		94	69.63		-0.09 (-0.22, 0.05)
Missing	1	0.49								
**Resident 65+ years-old**										
No	124	60.49		33	26.61		91	73.39		REF
Yes	81	39.51		24	29.63		57	70.37		-0.03 (-0.17, 0.11)
**Resident <18 years-old**										
No	162	79.02		50	30.86		112	69.14		REF
Yes	43	20.98		7	16.28		36	83.72		0.15 (0.00, 0.29)
**Resident with special needs**										
No	169	82.44		42	24.85		127	75.15		REF
Yes	32	15.61		14	43.75		18	56.25		-0.19 (-0.39, 0.01)
Missing	4	1.95								

**Evacuation knowledge, behavior, and perception of risks**

All residents living in Beaufort County, NC, were under either a voluntary (27.8%) or mandatory (72.2%) evacuation order for Hurricane Irene, but just over one quarter (27.1%) of the residents who were interviewed evacuated. Furthermore, over 35% of respondents did not recall any evacuation order for their home. Of the 72 respondents who knew that they were under some type of evacuation order, only 29.2% evacuated. It appears that in this study population, knowing about an evacuation order did not make a significant difference in a family’s decision to leave their home in preparation for Hurricane Irene. This may be due in part due to inadequate understanding of the evacuation order; of those who did correctly recall an evacuation order, many incorrectly thought it was voluntary, not mandatory (Table 2).

Evacuation decisions may be based primarily on individual perceptions of risk. In Beaufort County, NC, we found a dose-response association between level of perceived risk and evacuation; 38.8% of households who perceived they were at high risk evacuated, compared to 27.5% and 23.9% among households who perceived they were at medium and low risks, respectively. (Table 2)

**Table 2. Hurricane Irene evacuation behavior, knowledge, order status, and perception of risk in Beaufort County, North Carolina (n=205).** 

	**Study Population**			**Evacuated**			**Did Not Evacuate**	
	n	%		n	%		n	%
**Total**	205	100.00		57	27.80		148	72.20
**Actual evacuation order**								
Mandatory evacuation order	148	72.20		49	33.11		99	66.89
Voluntary evacuation order	57	27.80		8	14.04		49	85.96
**Knowledge of any evacuation order**								
Recalled any evacuation order	72	35.12		21	29.17		51	70.83
Did not recall any evacuation order	133	64.88		36	27.07		97	72.93
**Knowledge of evacuation order type (n=60)**								
Recalled mandatory evacuation order	27	45.00		9	33.33		18	66.67
Recalled voluntary evacuation order	33	55.00		7	21.21		26	78.79
**Perceived hurricane risk (n=192)**								
Low	92	47.92		22	23.91		70	76.09
Medium	51	26.56		14	27.45		37	72.55
High	49	25.52		19	38.78		30	61.22

**Social factors**

Nearly four out of five respondents had high social control (79.5%), with a median score of 6; 60.0% had high social cohesion, based on the two questions in the social cohesion score, with a median score of 6; 19.5% had high social cohesion due to markers of territoriality, with a median score of 0; 65.9% had high social capital, based on membership in organizations, with a median score of 1; 65.9% had high social capital due to social interactions, with a median of 5 visits per week or 15 “hellos” per day; and 52.0% had high social capital through volunteerism. None of the measures of social control, social capital, or social cohesion produced statistically significant RD estimates for failure to evacuate, though social capital measured by the number of social interactions was nearly significant at an alpha of 0.05 (RD = -0.12; 95% CI: -0.25, 0.01). (Table 3)

**Table 3. Social factors and the risk of failing to evacuate from Hurricane Irene in Beaufort County, North Carolina (n=205).** 

	**Study Population**			**Evacuated**			**Did Not Evacuate**			**Risk Difference** ** (95% Confidence Interval)**
	n	%		n	%		n	%		
**Social Control Score**										
<6	42	20.49		12	28.57		30	71.43		REF
6	163	79.51		45	27.61		118	72.39		0.01 (-0.16, 0.18)
**Social Cohesion Score**										
<6	82	40.00		24	29.27		58	70.73		REF
6	123	60.00		33	26.83		90	73.17		0.02 (-0.11, 0.16)
**Social Cohesion:Markers of Territoriality**										
None	165	80.49		48	29.09		117	70.91		REF
1 or more	40	19.51		9	22.50		31	77.50		0.07 (-0.10, 0.23)
**Social Capital: Organizations**										
None	70	34.15		19	27.14		51	72.86		REF
1 or more	135	65.85		38	28.15		97	71.85		-0.01 (-0.15, 0.13)
**Social Capital:Social Interaction**										
<15 hellos per day &< 5 visits	70	34.15		14	20.00		56	80.00		REF
≥15 hellos per day or≥ 5 visits	135	65.85		43	31.85		92	68.15		-0.12 (-0.25, 0.01)
**Social Capital: Volunteerism**										
No	98	47.80		26	26.53		72	73.47		REF
Yes	106	51.71		31	29.25		75	70.75		-0.03 (-0.16, -0.11)
Missing	1	0.49								

**Interaction between demographic groups and social factors**

Previous findings 11 suggest that exploring the interaction between demographic groups and social factors may show EMM, resulting in statistically significant point estimates and/or significantly different point estimates between stratified groups. As such, the interactions between demographic groups and social factors were assessed, and key findings are shown in Figure 2. Full results are presented in Appendix I.

Significant EMM was present for homes with special needs residents in relation to both social capital via social interactions (among no special needs residents, RD = -0.24, 95% CI: -0.37, -0.11; among special needs residents, RD = 0.44, 95% CI: 0.05, 0.84) and social cohesion via markers of territoriality (among no special needs residents, RD = -0.01, 95% CI: -0.19, 0.17; among special needs residents, RD = 0.50, 95% CI: 0.17, 0.83).

Evidence of potentially significant EMM was present between gender of respondent and social capital via organization membership (among male respondents, RD = 0.17, 95% CI: -0.01, 0.41; among female respondents, RD = -0.14, 95% CI: -0.31, 0.03), elderly residents and social cohesion via markers of territoriality (among homes with no elderly residents, RD = -0.05, 95% CI: -0.28, 0.19; among homes with elderly residents, RD = 0.23, 95% CI: 0.00, 0.46), and race/ethnicity and social cohesion via markers of territoriality (among white respondents, RD = 0.03, 95% CI: -0.16, 0.22; among non-white respondents, RD = 0.24, 95% CI: 0.04, 0.45). For the purposes of this study, the term “potentially significant EMM” implies that the 95% CIs overlap but do not contain the opposite strata’s point estimate 21. (Figure 2)

**Key stratified risk differences plots for failing to evacuate from Hurricane Irene in Beaufort County, North Carolina.**
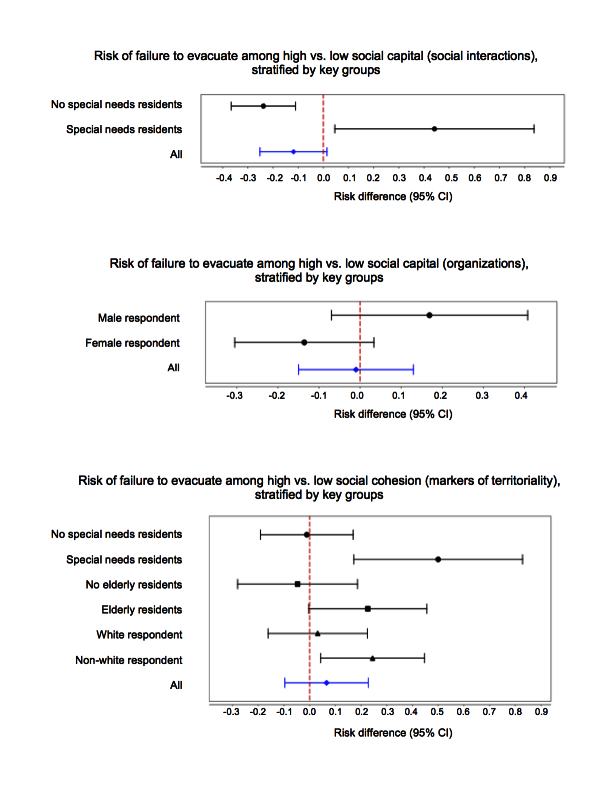

## Discussion and Conclusions

If there is evidence that social factors modify the associations between demographic characteristics and failure to evacuate from hurricanes and other natural disasters, these relationships should be considered during hazard planning to create tailored communication about evacuation to at-risk groups 22. In Beaufort County, NC, there is evidence of such interaction.

Furthermore, previous studies have shown that younger males have made up the majority of deaths from flooding associated with hurricanes in several recent storms, including Hurricanes Andrew and Floyd 23 ^,^ 24. If there are known interactions between gender or age and other social factors, such as social capital – as is evident for Beaufort County during Hurricane Irene – this high-risk group can be targeted with tailored evacuation messages through the organizations they belong to, such as churches, volunteer groups, community service groups, civic groups, veteran organizations, etc. Especially in light of the poor evacuation order knowledge among Beaufort County residents, tailored evacuation communication might be a low-cost solution to increase evacuation among high-risk groups by modifying their perception of risk, which could decrease overall hurricane-related morbidity and mortality.

Similar arguments can be made for a number of demographic and social interactions, such as households with children and social capital, gender and volunteerism, etc. However, one main limitation of this study is the low sample size, which provides limited statistical power to detect differences between stratified groups. It’s likely that additional EMM was present in this study population but went undetected. Future studies of this population should increase enrollment substantially to determine if additional meaningful interactions exist in Beaufort County, NC.

As is evident by the many individual demographic and social factors that are unassociated with failure to evacuate in this study, missing key interactions between at-risk groups and social factors masks the true influence of social and demographic factors on hurricane evacuation behavior. Due to the rapid response nature of the survey, we were unable to assess all possible effect measure modifiers but feel there is evidence that such interactions between other social and demographic variables could exist. As such, studies of evacuation behavior should focus on identifying all possible effect measure modifiers, especially among groups that have been previously identified as high-risk for disaster impact, such as individuals who: are living below poverty, are without a high school diploma, do not speak English well, do not have access to a vehicle, are racial/ethnic minorities; households with: persons 65+ years old, persons under 18 years old, persons with a disability, single-parent families; or individuals living in: mobile homes, multi-unit dwellings, crowded conditions, or group quarters 25.

Furthermore, understanding the social mechanisms behind these associations would lend even greater power to hazard planning efforts to encourage evacuation. If, for example, there is evidence that homes with a special needs resident may be less willing to evacuate because they feel that their ties to organizations, neighbors, friends, and the community (social capital) will provide protection and support during and after a disaster, evacuation messages could be crafted to address this scenario. However, this cross-sectional study is not able to provide any evidence for causation, only associations. Future studies should address this limitation.

Though this is a cross-sectional study, risk differences are reported due to the temporality of events in this study. Because the survey was conducted nearly 6-weeks after Hurricane Irene, it is unlikely that factors influencing social capital, social cohesion, and social control had changed dramatically; therefore, we have assumed that all social and demographic factors were present prior to making an evacuation decision.

Since Hurricane Irene made landfall 6 weeks prior to the survey, recall bias could have been a factor in this study. As a hurricane is a major event in the life of a community, it seems unlikely that residents would have trouble remembering the actions they took in response to the storm after such as short time. However, there is a possibility that those who evacuated or experienced damage may differentially recall more details about evacuation warnings compared to others who were not directly impacted or did not feel the need to evacuate.

In addition, an initial concern about selection bias due to missing data from residents whose homes had been destroyed in the hurricane is not a major limitation. Volunteers reported interviewing a number of residents at their property while rebuilding or waiting for the Federal Emergency Management Association to assess the damage, even though the property was not livable. Furthermore, volunteers were able to obtain 97.6% of the intended 210 interviews, which suggests that a representative sample of residents was available in each of the selected census blocks. In addition, despite differences in the proportion of women and older adults in our sample compared to the overall county, as neither age nor gender was found to have significant associations with evacuation in this study, we are confident that the findings are generalizable to Beaufort County, NC.

Finally, because this was a quick response study, the number of questions was limited to facilitate rapid data collection. As such, we were limited to 2 questions to assess social control, 3 questions to assess social cohesion, and 4 questions to assess social capital. Future studies might benefit from additional measures of these social factors.

In Beaufort County, NC, there is evidence that social factors modified the association between demographic characteristics and failure to evacuate from Hurricane Irene. As such, special attention should be given to households with special needs residents with high social capital or social cohesion, males with high social capital, elderly residents with high social cohesion, and non-white residents with high social cohesion when planning future disaster evacuation communications for residents of high risk coastal counties. Especially in light of findings on the importance of perceived risk, targeted, tailored messages could have a larger impact for these specific groups.

## Competing Interests

The authors declare that no competing interests exist.

## Appendix 1

### Interaction between social factors and demographic groups on the risk of failing to evacuate from Hurricane Irene (n=205).

**Table:**

	**No kids (n=162)**	** **	**Kids (n=43)**
**Resident under 18 years old**	RD (95% CI)		RD (95% CI)
Social control score	-0.07 (-0.25, 0.12)		0.32 (-0.15, 0.78)
Social cohesion score	0.07 (-0.09, 0.24)		-0.12 (-0.38, 0.14)
Social cohesion - Markers of territoriality	0.07 (-0.12, 0.26)		0.05 (-0.29, 0.38)
Social capital - Organizations	-0.04 (-0.20, 0.13)		0.12 (-0.16, 0.40)
Social capital - Social interaction	-0.13 (-0.28, 0.03)		-0.21 (-0.41, 0.00)
Social capital - Volunteerism	-0.05 (-0.20, 0.11)		0.01 (-0.27, 0.28)
			
	**No elderly (n=124)**	** **	**Elderly (n=81)**
**Resident 65+ years old**	RD (95% CI)		RD (95% CI)
Social control score	0.07 (-0.15, 0.29)		-0.10 (-0.38, 0.19)
Social cohesion score	0.05 (-0.12, 0.22)		-0.01 (-0.25, 0.24)
Social cohesion - Markers of territoriality	-0.05 (-0.28, 0.19)		0.23 (0.00, 0.46)
Social capital - Organizations	0.05 (-0.13, 0.22)		-0.11 (-0.36, 0.14)
Social capital - Social interaction	-0.15 (-0.32, 0.02)		-0.09 (-0.31, 0.13)
Social capital - Volunteerism	0.01 (-0.17, 0.18)		-0.09 (-0.31, 0.14)
			
	**No special needs (n=169)**	** **	**Special needs (n=32)**
**Resident with special needs**	RD (95% CI)		RD (95% CI)
Social control score	0.05 (-0.14, 0.23)		-0.19 (-0.67, 0.28)
Social cohesion score	-0.01 (-0.16, 0.14)		0.14 (-0.26, 0.55)
Social cohesion - Markers of territoriality	-0.01 (-0.19, 0.17)		0.50 (0.17, 0.83)
Social capital - Organizations	0.05 (-0.10, 0.20)		-0.13 (-0.65, 0.40)
Social capital - Social interaction	-0.24 (-0.37, -0.11)		0.44 (0.05, 0.84)
Social capital - Volunteerism	-0.08 (-0.22, 0.06)		0.11 (-0.31, 0.54)
			
	**White (n=148)**	** **	**Non-white (n=56)**
**Race/ethnicity**	RD (95% CI)		RD (95% CI)
Social control score	0.01 (-0.21, 0.23)		0.05 (-0.24, 0.34)
Social cohesion score	0.01 (-0.16, 0.18)		0.13 (-0.12, 0.37)
Social cohesion - Markers of territoriality	0.03 (-0.16, 0.22)		0.24 (0.04, 0.45)
Social capital - Organizations	0.00 (-0.17, 0.16)		-0.01 (-0.27, 0.26)
Social capital - Social interaction	-0.12 (-0.29, 0.04)		-0.07 (-0.32, 0.18)
Social capital - Volunteerism	0.01 (-0.15, 0.18)		-0.04 (-0.34, 0.25)
			
	**Male (n=73)**	** **	**Female (n=131)**
**Gender**	RD (95% CI)		RD (95% CI)
Social control score	-0.01 (-0.30, 0.28)		0.03 (-0.19, 0.24)
Social cohesion score	0.05 (-0.12, 0.30)		0.02 (-0.15, 0.19)
Social cohesion - Markers of territoriality	0.00 (-0.36, 0.37)		0.08 (-0.11, 0.27)
Social capital - Organizations	0.17 (-0.07, 0.41)		-0.14 (-0.31, 0.03)
Social capital - Social interaction	-0.15 (-0.39, 0.08)		-0.09 (-0.26, 0.08)
Social capital - Volunteerism	-0.12 (-0.36, 0.13)		0.04 (-0.12, 0.21)

## Appendix 2

### Survey Instrument

Download PDF
